# Molecular Characteristics of Uveal Melanoma: Insights from the Cancer Genome Atlas (TCGA) Project

**DOI:** 10.3390/cancers11081061

**Published:** 2019-07-27

**Authors:** Mathieu F. Bakhoum, Bita Esmaeli

**Affiliations:** 1 Shiley Eye Institute, Jacobs Retina Center, Viterbi Family Department of Ophthalmology, University of California San Diego, La Jolla, CA 92093, USA; 2 Orbital Oncology and Ophthalmic Plastic Surgery, Department of Plastic Surgery, The University of Texas MD Anderson Cancer Center, Houston, TX 77030, USA

**Keywords:** uveal melanoma, TCGA, whole exome sequencing, RNA seq, methylation, immune infiltrates

## Abstract

The Cancer Genome Atlas (TCGA) uveal melanoma project was a comprehensive multi-platform deep molecular investigation of 80 uveal melanoma primary tissue samples supported by the National Cancer Institute. In addition to identification of important mutations for the first time, it identified four different clusters (subgroups) of patients paralleling prognosis. The findings of the TCGA marker paper are summarized in this review manuscript and other investigations that have stemmed from the findings of the TCGA project are reviewed.

## 1. Background and Historical Perspective

Uveal melanoma (UM), the most common primary intraocular tumor in adults, is characterized by marked variability in its ability to metastasize. Up to half the patients with UM develop distant metastases, most commonly to the liver [1,2,3]. Once metastasis is clinically detected, the median survival is less than 12 months [2,4]. The Collaborative Ocular Melanoma Study (COMS), a large multi-center prospective trial, showed that local radiotherapy or enucleation are equally effective in achieving local control of the primary tumor with no difference in incidence of metastatic disease. Despite achieving local control of the primary tumor, overall survival rates have remained constant over the last four decades [5]. It is thought that micro-metastases may have already occurred by the time of enucleation, and that the chance of developing clinically significant metastases largely depends on the genetic underpinnings of the primary tumor. This variable metastatic proclivity was first demonstrated by Callender, who noted that UM with a predominant epithelioid cellular morphology, characterized by large nuclei, have worse prognosis than those with a predominantly spindle morphology [6,7]. Recurrent cytogenetic abnormalities have also been noted in UM and were found to correlate with metastatic tendencies. For instance, loss of chromosome 3 (monosomy 3) and gain of chromosome 8q are strong predictors of metastasis, whereas gain of 6p confers a better prognosis [8,9,10,11,12,13]. These non-random karyotypic aberrancies indicated that tumors with worse prognosis may have a unique molecular profile. In fact, UM with monosomy 3 were found to have a distinct gene expression profile [14]. This led to the development of a clinical prognostic test which has the ability to stratify tumors, solely based on gene expression patterns, into two major classes, with strikingly different survival outcomes [15,16].

The Cancer Genome Atlas (TCGA) project was a multi-center institutional effort, supported by the National Institute of Health, with the aim of providing a comprehensive genetic analysis of different cancers and establishing correlations with clinical outcomes. Eighty primary UM, with their associated clinicopathologic data, were included in a Rare Tumor Project of TCGA [17]. Mutations, genomic copy number alterations, transcriptomic and methylation profiles were analyzed for all eighty specimens. Additionally, whole genome sequencing was performed on fifty specimens and reverse-phase protein array (RPPA) was performed on eleven specimens. This review focuses on the main findings and analysis generated from TCGA, which was reported by Robertson et al. [17]. 

## 2. Mutational Landscape 

**Figure 1:**
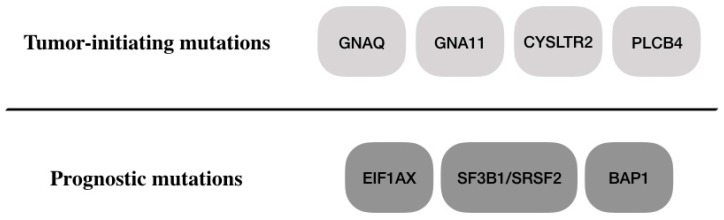
Two layers of mutations in uveal melanoma (UM). Mutations in genes involved in G-protein signaling are present in 98% of samples in a nearly mutually exclusive manner. They are also present in benign choroidal nevi and are therefore thought to occur in the earlier stages of tumor formation. A second layer of mutations occurs in EIF1AX, SF3B1, SRSF2, and BAP1. They are found in 66% of samples in a nearly mutually exclusive manner as well. These mutations confer different prognoses.

Unlike cutaneous melanoma, the mutational burden in UM is low. The median somatic mutation density in the TCGA cohort was found to be 1.1 per Mb in UM versus 18 per Mb in cutaneous melanoma [17,18]. There was no evidence of UV radiation mutational signature in UM consistent with prior observations [19]. Nine genes were significantly mutated in the cohort—*GNAQ*, *GNA11*, *BAP1*, *SF3B1*, *EIF1AX*, *CYSLTR2*, *SRSF2*, *MAPKAPK5*, and *PLCB4* [17], as shown in Table 1. Mutations in alpha G-protein subunits, *GNAQ* and *GNA11*, were found in 92.5% of the samples, in a mutually exclusive pattern consistent with prior observations [17,20,21,22]. Tumors that did not harbor these mutations were found to have mutations in *CYSLTR2*, a G-protein-coupled receptor, in 4% of samples and in *PLCB4*, a downstream effector of *GNAQ* signaling in 2.5% of samples [17,19,23], highlighting the involvement of G-protein signaling in the biology of uveal melanoma. There was no difference in outcomes (overall survival or metastasis) between these mutations. This suggests that activating mutations in *GNAQ/11* are an early event in UM oncogenesis. In fact, mutations in *GNAQ/11* were found in most benign nevi [20,24,25]. In vitro studies have supported the potential role of activating *GNAQ/11* mutations in cellular growth. *GNAQ/11.Q209L* mutation was shown to activate Yes-associated protein (*YAP*) and transcriptional coactivator with PDZ-binding motif *(TAZ)*, two transcriptional enhancers with oncogenic potential [26,27,28]. Ectopic expression of the activating mutation *GNAQ/11.Q209L* in zebrafish along with inactivation of the tumor suppressor *TP53* led to tumor formation [29,30], while expression of *GNAQ/11.Q209L* alone led to profound pigmentation defects without oncogenic transformation, supporting the hypothesis that the activating *GNAQ/11* mutations are precursor events requiring a ‘second hit’ in order to lead to malignant transformation, as shown in Figure 1 [31].

A second layer of mutations consist of mutations in *SF3B1*, *EIF1AX*, *BAP1*, and were found in a nearly mutually exclusive manner. In contrast to mutations in genes involved in G-protein signaling, these mutations were associated with markedly distinct prognoses. Samples with *BAP1* mutations (33% of the cohort) had unique somatic copy number alterations (SCNA) and gene expression profiles and were associated with poor survival outcomes. All tumors with *BAP1* mutations had genomic copy loss in chromosome 3, while none of those with *EIF1AX* and only 22% of UM with *SF3B1* mutations had monosomy 3. Somatic mutations in *BAP1*, located on 3p21.1, were previously shown to confer a strong predilection for metastasis [17,32,33]. In the TCGA cohort, single nucleotide polymorphism/insertion-deletion (SNP/indel) analysis of monosomy 3 whole exome sequencing (WES) initially identified *BAP1* mutation in 40.5% of monosomy 3 samples. RNA/DNA assembly-based methods led to the identification of additional BAP1 alterations, raising the percentage to 83.3% in UM with monosomy 3, in line with other reports [17,32]. It is possible that deeper whole genome sequencing with longer reads, which was not performed, could have detected additional *BAP1* alterations in UM with monosomy 3. *EIF1AX* and *SF3B1* mutations were present in 35% of samples.

Despite the strong association with worse outcomes, a mechanistic link between *BAP1* loss and highly aggressive UM remains elusive. BAP1, which was initially identified in a yeast 2-hybrid screen as a protein that interacts with BRCA1 [34], is a deubiquitinating enzyme. BAP1-mediated deubiquitination of γ-tubulin and MCRS1 (microspherule protein 1) has been shown to contribute to chromosomal stability [35,36]. BAP1 has also been implicated in DNA double-strand break repair [37,38]. Interestingly, in the TCGA report, genes involved in DNA damage repair/response (DDR) were upregulated in monosomy 3 tumors harboring *BAP1* mutations compared to disomy 3 tumors with *SF3B1* mutations [17].

Mutations in *SRSF2* were found in three samples which had wildtype *EIF1AX* and *SF3B1*. Samples with *SRSF2* mutations had an SCNA profile similar to that of *SF3B1*, suggesting a common molecular basis for both genes in the UM development. *SRSF2* gene product is a member of the serine/arginine-rich family of pre-mRNA splicing factors, and *SF3B1* encodes subunit 1 of the splicing factor 3b protein complex. In fact, UM with mutations in either of those genes had alternatively spliced transcripts in a subset of genes compared to UM with wildtype *SF3B1/SRSF2*. 

## 3. Patterned Aneuploidy 

Aneuploidy denotes a genomic state where there is an aberrant number of chromosomes, i.e., a non-euploid state. Aneuploidy is very prevalent in cancer and is thought to arise from chromosomal missegregation during mitosis [41,42,43,44,45]. Consequently, genomic heterogeneity can facilitate tumor evolution [44,46]. In the TCGA-UM cohort, chromosomal alterations were inferred from whole exome sequencing and SNP microarrays. Unsupervised clustering of all eighty samples showed that they segregate in four distinct clusters with different prognosis. Clusters 1 and 2 retained both copies of chromosome 3 while clusters 3 and 4 were mainly characterized by monosomy 3. Clusters 1 and 2, which had better prognoses, were enriched for 6p gain. Expectedly, *BAP1* mutations were limited to clusters 3 and 4, which were defined by monosomy 3. *EIF1AX* mutations were only present in cluster 1, and mutations in *SF3B1/SRSF2* were mainly present in cluster 2 and, to a lesser extent, in cluster 3. Gain of 8q was present in clusters 2, 3, and 4, with the latter cluster having high copy gain or amplification of 8q. Essentially, while 8q gain was seen in a subset of UM with disomy 3, nearly all samples with monosomy 3 had gain or amplification of 8q. Moreover, isodisomy 8 was seen in a subset of UM with monosomy 3—these had the worst prognosis. These recurrent chromosomal alterations were in agreement with prior observations [9,10,11,12,47,48,49,50,51,52]. Other karyotypic abnormalities appeared more random but were more abundant in cluster 4 (monosomy 3/*BAP1*-aberrant/8q amplification) while virtually absent in cluster 1 (disomy 3/*BAP1*-competent). Whole genome doubling was also noted in tumors with monosomy 3. These associations hint to a possible role for *BAP1* loss in inducing chromosomal instability. However, it must be cautioned that chromosomal instability and aneuploidy are not synonymous. The former is a dynamic state caused by ongoing errors in chromosome segregation, while the latter is a characterization of a static state, which in many cases may result from ongoing chromosomal instability [41]. Selective genomic copy numbers may also occur independent of chromosomal missegregation. For instance, extrachromosomal DNA segments may lead to amplification of genomic regions containing oncogenes [53]. Thus, it should be pointed out that recurrent chromosomal abnormalities in UM do not necessarily indicate higher levels of chromosomal instability. For instance, UM with 6p gain is aneuploid by definition, but may have a lower level of chromosomal instability than UM with monosomy 3 and 8q gain.

Given that *BAP1* mutations were always present in the context of monosomy 3, Robertson et al. analyzed cancer cell fractions to estimate the relative timing of *BAP1* loss versus monosomy 3 in the TCGA uveal melanoma cohort [17]. Cancer cell fractions of monosomy 3 were close to 1 (mean 0.97), whereas those of *BAP1* alterations were lower (mean 0.88), and other passenger mutations on chromosome 3 occurred with much less frequency (mean 0.60). This suggests that loss of chromosome 3 occurs prior to *BAP1* alterations which occurs at a higher frequency than other passenger mutations located on chromosome 3. 

## 4. Epigenetic Fingerprints

Another means by which cancer cells may up- or downregulate gene expression is through DNA methylation. CpG islands are CG-rich regions where a cytosine nucleotide can be methylated by DNA methyltransferases to form 5-methylcytosine. Promoter methylation often leads to gene silencing. In cancer, hyper/hypo-methylation is common, and is a means by which a cell can silence or upregulate genes [54]. TCGA provided, for the first time, a comprehensive analysis of DNA methylation profiles of UM using the Infinium HumanMethylation450 BeadChip Kit (Illumina), a microarray-based test which surveys 450,000 methylation loci. Clustering of the most variable 1% of these probes (approximately 4500 probes) revealed that monosomy 3/BAP1-aberrant tumors had a unique methylation profile. Within disomy 3 UM, tumors with *EIF1AX* mutations had a different methylation profile than those with *SF3B1* mutations. This clustering was similar to that of SCNA, suggesting an epigenetic role in the evolution of UM. 

## 5. Transcriptomic Analysis

Unbiased clustering of mRNA transcripts revealed four clusters. Expectedly, monosomy 3/*BAP1*-aberrant tumors had a distinct transcriptomic profiles than tumors with disomy 3 and high BAP1 levels. These two main clusters also correlated with the prognostic twelve-set marker genes that are commonly used in clinical prognostication [15]. Each cluster could be further subdivided into two groups. Unbiased clustering of long noncoding (lncRNA) followed the same patterns as mRNA. LncRNAs CYTOR, PVT1, and BANCR, which are associated with other solid cancers [55,56], were more abundant in the poor-prognosis clusters 3 and 4. PVT1, which has been shown to be associated with MYC protein stabilization [57]. Clusters based on microRNA (miRNA) were slightly different than mRNA or lncRNA subtypes.

Differential gene expression analysis revealed that the poor-prognosis mRNA cluster 4 was enriched for immune genes and genes located on 8q. Unlike many other malignancies, including cutaneous melanoma, the presence of an immune infiltrate and increased expression of human leukocyte antigens (HLA) in UM has been associated with poor prognosis [58,59]. For instance, immunohistochemical analysis showed that tumors with high lymphocytes (containing more than 100 lymphocytes per 20 high power field (HPF)) correlated with a 15 year survival rate of 37%, as opposed to 70% when the tumors had less than 100 lymphocytes per 20 HPF [60]. Similarly, tumors with higher immune infiltration were associated with worse prognostic factors, such as monosomy 3, and patients had higher rates of metastasis [58,59,60,61,62,63]. In the TCGA cohort, the frequency of an immune infiltrate was inferred by DNA methylation and RNA-seq, and was estimated to constitute 30% of monosomy 3 UM while absent in disomy 3 UM. Transcriptomic analysis showed enrichment of HLA genes and others involved in interferon-γ signaling, T cell invasion, cytotoxicity, and immunosuppression in high-risk tumors. This finding correlated with a prior analysis that was based on whole genome sequencing of UM [52]. Dissecting this unique immune infiltrate is significant because metastatic UM do not respond to immune checkpoint inhibitors (see below). 

## 6. Pathway Discovery

**Figure 2:**
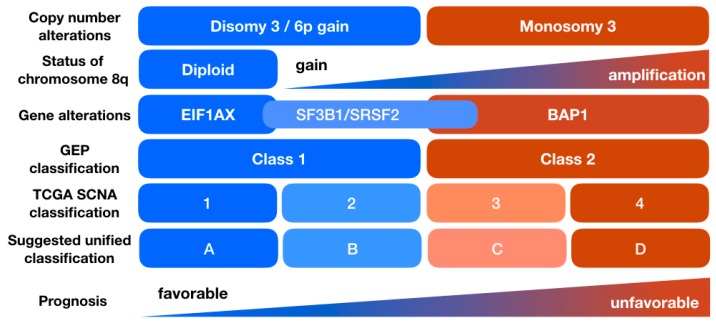
An overview of UM subtypes. Prognostic classes can be inferred from either somatic copy number alterations (SCNA), gene alterations, or gene expression. The A, B, C, and D classification was suggested to avoid confusion between the numerical classification used in both the Cancer Genome Atlas (TCGA) and the commercially available test (GEP classification).

Clustering of UM based on their SCNA, methylation, mRNA, miRNA, or lncRNA profiles led to four major clusters in each instance, as shown in Figure 2. Monosomy 3 UM had distinct genomic, transcriptomic, and epigenetic profiles than disomy 3 UM. Both disomy 3 and monosomy 3 UM could be further subdivided into two subgroups based on their SCNA profiles, with a more refined prognosis. Transcriptomic clustering allowed subdividing disomy 3 and monosomy 3 UM into two distinct groups as well, albeit this clustering was not fully concordant with SCNA clustering. Nevertheless, subgrouping into distinct clusters with different outcomes insinuate a biological basis behind different metastatic tendencies within high-risk tumors. In order to elucidate possible pathways associated with metastasis, Robertson et al. applied PARADIGM to infer subset-specific pathways from multi-dimensional genomics data. This led to the identification of four major and one minor groups [17]. Clusters 3 and 4 were *BAP1*-aberrant/monosomy 3 tumors. Cluster 4 was enriched for genes involved in DNA damage repair/response (DDR) and hypoxia. MYC signaling was also highest in cluster 4. MYC is an oncogenic transcription factor expressed on the long arm of chromosome 8, which is also amplified in cluster 4. It can complex with MAX and/or MIZ1. Robertson et al. found that targets of MYC/MAX/MIZ were represented in cluster 4. Interestingly, MYC/MAX complex activity levels were high in tumors with no 8q gain indicating that other factors, in addition to 8q gain, may enhance MYC signaling. Additional genes upregulated in the high-risk cluster 4 included *JUN*-*FOS* and *JAK2*-*STAT1/3*, both of which are implicated in multiple malignancies. *JAK*-*STAT* signaling can alter transcription driven by extracellular ligand binding, including cytokines [64,65]. Consistently, cluster 4 tumors were enriched for immune-related genes. Cluster 3 tumors on the other hand were enriched in genes involved in cell cycling and cellular proliferation, with low enrichment in genes involved in DDR and hypoxia, when compared to cluster 4. Even though both clusters had monosomy 3 and low *BAP1* levels, they had different signaling profiles. Elucidating biological pathways associated with worse outcomes (cluster 4 versus 3) can provide insight into biological pathways that drive tumor evolution. 

## 7. Clinical Prognostic Tests

Clinical prognostication is important for many reasons. Foremost, patients often want to know their prognosis. Second, tumor prognostic classification can modify the frequency of metastatic surveillance in the various prognostic groups. Third, tumor stratification can help identify high-risk patients that may be appropriate for enrollment in clinical trials for adjuvant therapy. TCGA was the largest comprehensive study that concurrently evaluated multiple prognostic parameters and associated them with clinical outcomes. In order to avoid confusion between TCGA and the commercial Castle Biosciences classification (Class 1A, 1B, or 2), Jager et al. in a review manuscript based on TCGA findings suggested labelling TCGA-clusters using an alphabetical nomenclature—A, B, C, and D for clusters 1, 2, 3, and 4, respectively [66]. Vichitvejpaisal et al. applied a simplified TCGA classification on 658 UM samples from fine needle aspiration and validated TCGA findings in a large retrospective cohort [67]. Class A was defined as disomy 3 tumors, class B as disomy 3 tumors with 8q gain, class C as monosomy 3 tumors with 8q gain, and class D tumors as monosomy 3 tumors with 8q amplification. It must be cautioned however that the TCGA classification hinges on the platform analyzed (RNA vs. DNA). As we mentioned, clusters 1 and 2 can be distinguished from clusters 3 and 4 using any platforms. However, assigning a disomy 3 UM sample to cluster 1 versus 2, or a monosomy 3 UM sample to cluster 3 versus 4, may differ depending on the platform used. We suggest that when referring to TCGA classification, the platform should be clearly indicated.

In the United States, ocular oncologists utilize either a DNA-based test which relies on karyotypic analysis of the tumor (mainly monosomy 3, 8q, and 6p gain) or a RNA-based test, commercially offered by Castle Biosciences, wherein a 12-gene panel is used to estimate prognosis (Class 1A, 1B, or 2, with low, intermediate, and high risk of metastasis, respectively). In Europe, chromosomal analysis is more prevalent. The multi-dimensional TCGA-UM analysis indicated that segregating tumors into two major prognostic groups can be equally achieved by analyzing chromosome 3 status (disomy 3 vs. monosomy 3) or analyzing the transcriptome (Castle Biosciences GEP class 1 vs. 2/TCGA clusters 1 and 2 vs. 3 and 4). In other words, clusters 3 and 4 (Castle Biosciences GEP class 2) reflect the transcriptome of monosomy 3 tumors. While both approaches (RNA or DNA-based) may identify identical tumors, different tests may have different sensitivities, specificities, and limitations. For instance, chromosome 3 status can be inferred from whole genome sequencing (WGS), WES, SNP, multiplex ligation-dependent probe amplification (MLPA), or fluorescence in situ hybridization (FISH). Gene expression profiles rely on comparing relative levels of RNA from a 12-gene panel. This test’s major drawback is its inability to distinguish melanoma cells from other choroidal tumors [68]. It is likely that test-specific limitations may account for reported discrepancies in survival data reported in various studies, rather than superiority of one approach over another. These inherent variations must be taken into consideration when reporting survival or metastasis outcomes, or enrolling patients in prospective trials for adjuvant therapy. It is also important to note that even though transcriptomic clustering segregated disomy 3 tumors into two subgroups, there was no evidence that these groups correlated with Castle class 1 subgroups (1A versus 1B). In addition, the TCGA SCNA analysis allowed for the first time the identification of two different groups with different outcomes in tumors with monosomy 3—a distinction that was not identified through prior prognostication methods, as shown in Figure 2.

## 8. Therapeutic Implications

UM can be treated by iodine or ruthenium plaque radiotherapy, proton beam radiation therapy, or enucleation. These interventions, albeit successful in controlling the primary tumor and reducing recurrence, do not reduce the chance of metastasis. Ideally, targeted therapies would be effective against the primary tumor as well as micrometastases. Activating mutations in *GNAQ/11* are prevalent in UM, and are also found in choroidal nevi [20,24,25]. In the TCGA cohort, mutations in *GNAQ/11*, *CYSLTR2,* and *PLCB4* (2.5%) were found in 92.5%, 4%, and 2.5% of the samples, respectively, highlighting the involvement of G-protein signaling in the biology of uveal melanoma. In essence, inhibiting *GNAQ/11* or their downstream signaling appears as a promising approach. However, targeting *GNAQ/11* downstream effectors have not led to successful outcomes in metastatic UM [69,70]. Direct inhibition of oncoproteins Gαq/11 may be a better alternative. This can be achieved by a selective pharmacologic inhibitor FR900359 (FR), which has shown efficacy in pre-clinical models [71,72,73,74,75,76]. Interestingly, even though *GNAQ/11* mutations were present in 93% of the samples, protein analysis showed that levels of PKC, a downstream effector of GNAQ/11, were markedly higher in monosomy 3/BAP1-aberrant UM, indicating that *GNAQ/11* signaling may be further enhanced in M3 tumors [17].

Immune checkpoint inhibitors have proven very beneficial in the management of metastatic cutaneous melanoma and in a few cases of metastatic conjunctival melanoma [77]. Yet, attempts to apply this approach in metastatic uveal melanoma have failed [70]. It is possible that the relatively low mutational burden, and consequently the paucity of neoantigens, may partly explain why metastatic UM do not respond to immune checkpoint inhibitors. However, it must be cautioned that the inverse relationship between tumor mutational burden and response to immunotherapy is variable across different tumor types [78]. Thus, there are likely other underlying factors in addition to a lower mutational burden that may explain UM’s resistance to immune checkpoint inhibitors. For instance, UM cells may secrete inhibitory ligands to dampen anti-tumor immunity. Transcript levels of *IDO1* and *TIGIT*, both of which are immune checkpoint inhibitors that can limit T cells’ cytotoxic effects, were high in monosomy 3 UM with high *CD8* levels. Robertson et al. postulate that combinations of agents targeting IDO1 and TIGIT along with CTLA-4 or PD1 inhibition may therefore prove beneficial [17].

## 9. Conclusions

In summary, the comprehensive multi-dimensional analysis by TCGA revealed that UM can be subdivided into four distinct groups with different outcomes rather than three prognostic groups previously reported using the commercially available RNA-based prognostic tests. TCGA findings allowed us to validate many prognostic markers currently used in clinical practice in a single cohort of patients using a comprehensive integrated approach. The data from the integrative pathway analysis in TCGA UM report are publicly available to investigators and this great resource provides a biological platform for developing effective targeted therapies for UM patients in the future. 

## Figures and Tables

**Table 1:** Genes frequently mutated in uveal melanoma. Source: cBioPortal [39,40].

Gene	Percentage of Samples with Mutations	Recurrent Alleles—Protein Change (Frequency)
**GNAQ**	50%	Q209P/L (90%) R183Q (5%) G48*/V (5%)
**GNA11**	45%	Q209L (94%) R183C (3%) R166H (3%)
**BAP1**	33%	Multiple *
**SF3B1**	23%	R625H/C (78%) K666T (11%) H662R (5%) T663P (5%)
**EIF1AX**	13%	G6D (20%) G8R (20%) G9R/D (20%) G15D (20%) W70R (10%)
**CYSLTR2**	4%	L129Q (100%)
**SRSF2**	4%	Multiple
**PLCB4**	2.5%	D630N/V/Y (100%)
**MAPKAPK5**	2.5%	Truncating mutations

* Missense, in-frame, and truncation mutations.
